# *SnRK*-*PP2C*-*PYL* Gene Families in *Citrus sinensis*: Genomic Characterization and Regulatory Roles in Carotenoid Metabolism

**DOI:** 10.3390/metabo15090610

**Published:** 2025-09-12

**Authors:** Pengjun Lu, Zhenting Shi, Tao Liu, Jianqiu Ji, Jing Li, Wentao Li, Chongbo Sun

**Affiliations:** 1 Innovation Center of Chinese Medicinal Crops, Zhejiang Academy of Agricultural Sciences, Hangzhou 310021, China; 2 Institute of Horticulture, Zhejiang Academy of Agricultural Sciences, Hangzhou 310021, China; 3 Hangzhou Raw Seed Growing Farm, Hangzhou 311115, China; 4 International Joint Laboratory for Agricultural Plant Metrology and Equipment Innovation, College of Life Sciences, China Jiliang University, Hangzhou 310018, China

**Keywords:** SnRK, PP2C, PYL, carotenoid, *Citrus sinensis*, gene family, metabolism

## Abstract

**Background/Objectives**: Carotenoids in citrus are vital nutritional compounds and precursors of the stress hormone abscisic acid (ABA). SNF1-related kinases (SnRKs)—key regulators of plant stress signaling that phosphorylate is targeting proteins for post-transcriptional regulation—mediate ABA signaling through its subfamily SnRK2-phosphatase type-2C (PP2C)-PYR1-LIKE (PYL) cascades. This study aims to identify the SnRK-PP2C-PYL family members and decipher their underlying post-transcriptional regulatory mechanisms which control carotenoid metabolism in *Citrus sinensis* for improved nutrition and stress resilience. **Methods**: SnRK, PP2C, and PYL were identified by integrated HMMER-blastp-CDD pipeline in the *Citrus* genome. Using two carotenoid-divergent cultivars, ‘Newhall’ (yellow) and ‘Cara Cara’ (red, hyperaccumulating linear carotenoids), we conducted spatiotemporal expression profiling and integrated transcriptomic and metabolomic data via Weighted Gene Co-expression Network Analysis (WGCNA) to identify modules correlated with accumulation. **Results**: We identified 26 *CsSnRKs* (1 *SnRK1*, 7 *SnRK2*, 18 *SnRK3*), 57 *CsPP2C*s, and 7 *CsPYL*s in *Citrus sinensis*. Despite a >26-fold difference in linear carotenoids, structural gene expression was similar among cultivars, strongly implicating post-transcriptional control. WGCNA identified a key turquoise module highly correlated with linear carotenoid content. This module contained phosphorylation-related genes (*CsSnRK1*/*3.5*/*3.6*/*3.16*, *CsPP2C14*/*15*/*33*/*35*/*38*/*40*/*43*/*56*, and *CsPYL6*), biosynthetic genes (*CsPSY1*, *CsZISO*, and *CsZDS*), and candidate transcription factors. Network analysis predicted that *CsSnRK*s, *CsPP2C*s, and *CsPYL*s regulate phytoene-derived carotenoid biosynthesis. **Conclusions**: We propose a novel phosphorylation-mediated post-transcriptional regulatory network in carotenoid accumulation. This mechanism bridges ABA signaling and metabolic adaptation, providing crucial molecular targets for engineering nutrient-dense and climate-resilient citrus varieties.

## 1. Introduction

**Figure 1:**
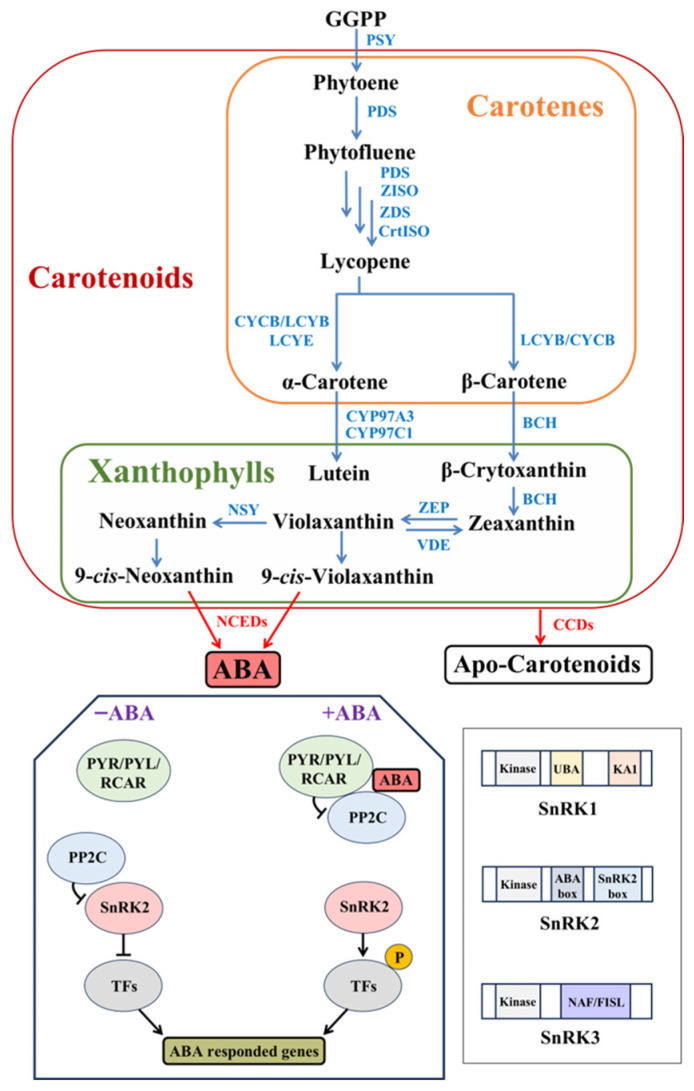
Carotenoid biosynthetic pathway in higher plants and ABA signaling via SnRK-PP2C-PYL cascades. Illustration depicts: (1) core carotenoid biosynthesis from geranylgeranyl diphosphate (GGPP) to α/β-branch carotenoids and ABA precursor cleavage by NCED; (2) ABA signal transduction through PYL receptor-PP2C phosphatase-SnRK2 kinase cascades; and (3) structural domains distinguishing SnRK subfamilies. GGPP, geranylgeranyl pyrophosphate; PSY, phytoene synthase; PDS phytoene desaturase; Z-ISO, ζ-carotene isomerase; ZDS, ζ-carotene desaturase; CrtISO, carotene isomerase; CYCB, chromoplast-specific lycopene β-cyclase; LCYB, lycopene β-cyclase; LCYE, lycopene ε-cyclase; BCH, β-carotene hydroxylase; ZEP, zeaxanthin epoxidase; VDE, violaxanthin de-epoxidase; NSY, neoxanthin synthase; NCED, 9-cis-epoxycarotenoid dioxygenase; CCD, carotenoid cleavage dioxygenase.

*Citrus sinensis* (also written as *Citrus × sinensis*) is a hybrid between the pomelo (*Citrus maxima*) and mandarin (*Citrus reticulata*), known as the cultivated sweet orange. *Citrus sinensis* stands as the most globally significant *Citrus* species in terms of cultivated area and production [1,2,3]. Its commercial value hinges critically on fruit pigmentation, primarily governed by carotenoids—tetraterpenoid pigments that confer vibrant coloration and serve as precursors for vitamin A, potent antioxidants with demonstrated roles in mitigating carcinogenesis, cardiovascular diseases, and neurodegeneration [4,5,6]. Beyond human health benefits, carotenoids underpin photosynthesis, photoprotection, and phytohormone synthesis [5]. Carotenoids include carotenes, which lack oxygen atoms, and xanthophylls, which contain oxygen. The carotenoid biosynthetic pathway is well-characterized [7] (Figure 1). Enzymes involved in carotenoid biosynthesis are primarily localized in plastids and encoded by nuclear genes. Phytoene synthase (PSY), the rate-limiting enzyme of the carotenoid pathway, catalyzes the condensation of two molecules of geranylgeranyl diphosphate (GGPP) to form the first carotenoid molecule, phytoene. Phytoene is then processed by several enzymes into lycopene [5]. The pathway then splits into α,β- and β,β-branches at lycopene, leading to degradation by carotenoid cleavage dioxygenase (CCD). Carotenoids can be cleaved into volatile compounds that serve as plant signaling molecules and aroma compounds, or they can be degraded into apocarotenoids. 9-*cis*-epoxycarotenoid dioxygenase (NCED), a member of the CCD family, specifically cleaves 9-*cis*-violaxanthin and 9-*cis*-neoxanthin to generate abscisic acid (ABA) [5,7].

Transcriptional regulation of carotenoid metabolism is increasingly understood. Light-responsive TFs (PIF1 repressing *PSY*/carotenoids in darkness; HY5 activating *PSY* under light/temperature) critically regulate carotenoid biosynthesis [8,9,10], while MADS-box TFs (e.g., tomato RIN/AGAMOUS-like1/FRUITFULL) directly control *PSY*/carotenoid levels during ripening [10,11]. However, the regulatory mechanisms, particularly post-transcriptional controls, governing carotenoid flux remain incompletely resolved. As central stress-signaling regulators, SNF1-related kinases (SnRKs) broadly coordinate post-translational responses via phosphorylation [12,13]; however, their specific modes of action in regulating carotenoid biosynthesis and degradation remain unresolved and demand systematic investigation. ABA, a carotenoid-derived sesquiterpenoid hormone, orchestrates plant development and stress responses [13,14]. A major breakthrough in ABA signaling was the identification of the core signaling components: the pyrabactin resistance (PYR)/ PYR1-like (PYL)/RCAR receptor family, phosphatase type 2C (PP2C), and SnRK2 kinases belong to a subfamily of SnRK [15,16,17]. The established model shows that ABA binding to PYR/PYL/RCAR induces conformational changes enabling receptor-PP2C interaction, which disrupts the PP2C-mediated inhibition of SnRK2s (Figure 1). This releases active SnRK2s to phosphorylate downstream targets, including transcription factors [18] (Figure 1). *SnRK*s comprise three subfamilies—*SnRK1* (energy sensing), *SnRK2* (ABA/osmotic stress), and *SnRK3* (Ca^2+^ signaling)—all sharing a conserved kinase domain but with subgroup-specific domains (KA1/UBA in *SnRK1*; NAF in *SnRK3*) [12,13] (Figure 1). In Arabidopsis, these families are represented by 14 PYR/PYL/RCAR receptors [17], 80 PP2Cs [19], and 38 SnRKs (3 SnRK1, 10 SnRK2, 25 SnRK3) [12]. ABA is a carotenoid-derived metabolite functioning as a phytohormone that regulates diverse physiological processes. It maintains multifaceted equilibrium during plant growth through hormonal crosstalk. As products shaped by extensive evolution, the carotenoid metabolic pathways in plants likely possess intricate feedback regulation mechanisms. A compelling scientific question worthy of exploration is the potential regulatory role of the SnRK, PYL, and PP2C protein families in carotenoid biosynthesis. Our preliminary research on the ‘Cara Cara’ mutant, which hyperaccumulates linear carotenoids in its juice vesicles, revealed that neither transcriptional differences in biosynthetic genes nor chromoplast sink capacity explain its enhanced accumulation. Thus, post-transcriptional regulation, particularly phosphorylation, is strongly implicated as the underlying mechanism [20,21].

Characterizing the temporal and spatial expression patterns of the *SnRK*, *PYL*, and *PP2C* family members during the development of these two sweet orange genotypes with distinct carotenoid accumulation phenotypes will provide crucial insights into this underlying regulatory mechanism. A 2012 *Citrus* study [22] identified the SnRK2 subfamily, PYL, and clade-A type-2C protein phosphatases (PP2CAs). However, it relied solely on BLASTp alignments with known Arabidopsis sequences. Likely due to limited genome data at the time, the number of identified *SnRK2*, *PYL*, and *PP2CA* genes differs from this study. Here, we comprehensively identify *SnRK*, *PYL*, and *PP2C* gene families in *Citrus sinensis* using an integrated HMMER-blastp-CDD pipeline, updating previous partial annotations. We further compare spatiotemporal expression of these genes in ‘Newhall’ (yellow pulp) and ‘Cara Cara’ (red pulp) across fruit development, integrating transcriptomic and carotenoid metabolite data. Weighted Gene Co-expression Network Analysis (WGCNA) employs eigengene network methodology to analyze gene expression correlation matrices, facilitating the identification of phenotype-associated gene modules and candidate biomarkers [23].

This study employs integrated transcriptomic and metabolomic co-expression analysis to construct correlation networks, identifying modules of co-expressed genes and highly connected regulatory hubs to explore phosphorylation-mediated post-transcriptional networks regulating carotenoid metabolism in sweet orange. Our objectives are (1) the identification of *SnRK*, *PYL*, and *PP2C* families in sweet orange; (2) expression profiling of these regulators in contrasting carotenoid-accumulating genotypes; and (3) co-expression network analysis to uncover putative regulatory nodes linking post-transcriptional regulation and ABA signaling to carotenoid metabolism. Our analysis sheds light on potential regulatory mechanisms and nominates prioritized candidate genes implicated in post-transcriptional control. These candidates provide a foundation for future experimental validation and represent potential molecular targets for precision citrus breeding aimed at enhancing nutritional carotenoid content.

## 2. Materials and Methods

### 2.1. Genome-Wide Identification, Characterization, and Phylogenetic Analysis of CsSnRK, CsPP2C, and CsPYL Genes

The Pfam domains PF00069 (SnRK) and PF00481 (PP2C) were downloaded from the InterPro Scan database (https://www.ebi.ac.uk/interpro/entry/pfam/, accessed on 1 June 2025). Since no established Pfam domain exists for PYL proteins, we constructed a custom hidden Markov model (HMM) profile using 14 *Arabidopsis thaliana* AtPYL protein sequences. The workflow involved performing multiple sequence alignments of 14 AtPYL peptides, building the HMM profile via hmmbuild, and generating the HMM file: 14AtPYLpep.stockholm. The software HMMER 3.0 [24] was used to perform local hmmbuild and hmmsearch on annotated protein sequences from *Citrus sinensis* (http://citrus.hzau.edu.cn, accessed on 1 June 2025) with a cut-off E-value of <1 × 10^−20^ for both full length and best domain. Protein sequences of AtSnRKs [12], AtPP2Cs [19], and AtPYLs [17] from Arabidopsis were downloaded from NCBI (https://www.ncbi.nlm.nih.gov/, accessed on 1 June 2025). A local BLASTP search was then performed using the protein AtSnRKs, AtPP2Cs, and AtPYLs sequences as query and annotated protein sequences from *Citrus sinensis* as the database, with default parameters, and was further filtered with a threshold of identity > 50%. The candidate sequence was further confirmed in the Conserved Domain Database (CDD) (https://www.ncbi.nlm.nih.gov/Structure/cdd/wrpsb.cgi, accessed on 1 June 2025) to remove the false-positive sequences.

The candidate protein sequences of AtSnRKs and CsSnRKs (Figure S1), AtPP2Cs and CsPP2Cs (Figure S2), and AtPYLs and CsPYLs (Figure S3) were separately performed for multiple sequence alignments using the CLUSTAL tool in MEGA 7.0 software [25] with default settings. Any sequences with poor alignment were excluded. The phylogenetic tree was constructed using the neighbor-joining method with 1000 bootstrap replicates in MEGA 7.0 software. To enhance the visual presentation of the tree, Evolview (http://www.evolgenius.info/evolview/, accessed on 1 June 2025) was utilized [26].

### 2.2. Plant Materials and Carotenoid/Chlorophyll Extraction with HPLC Quantification

‘Newhall’ is a cultivar derived from a mutation of the Washington navel orange. It is characterized by a relatively small, flat-round or round-headed canopy with short, dense branches and dark green leaves. The fruit is elliptical, relatively large, and has a smooth, orange-red to deep-orange rind. In contrast, the origin and pedigree of ‘Cara Cara’ remain unclear, though its main agronomical traits are largely consistent with those of ‘Newhall’. The ‘Newhall’ and ‘Cara Cara’ plant materials used in this study were collected from orchard-grown trees in Zhejiang Province, China. The leaves, flowers, and fruits were immediately frozen in liquid nitrogen and stored at −70 °C for subsequent analysis. All experiments in this study, including HPLC quantification, RNA-seq, and qPCR validation, were performed with three independent biological replicates (*n* = 3), and for carotenoids/chlorophylls extraction and measurement by HPLC, according to a method previously described [27]. Fresh tissues (0.4 g) were ground in liquid nitrogen and extracted with chloroform/methanol/Tris-HCl (pH 7.5). After centrifugation, the chloroform phases were collected and dried under nitrogen gas. The residue was saponified with 6% KOH in methanol at 60 °C for 0.5 h (this step was omitted for chlorophyll analysis). Water and chloroform were added to saponified mixtures, and after centrifugation, the chloroform phases were recollected and dried under nitrogen gas. HPLC analysis used a Waters Alliance 2695 system (Waters Corporation, Milford, MA, USA) with a YMC reverse-phase C30 column at 25 °C. Mobile phases comprised (A) methanol, (B) 80% methanol with 0.2% ammonium acetate, and (C) tert-butyl methyl ether. The gradient program was 95% A/5% B (0–6 min), linear transition to 80% A/5% B/15% C (7 min), held (7–12 min), transition to 30% A/5% B/65% C (32 min), held (32–48 min), return to 95% A/5% B (50 min), and held to 60 min. Quantification used peak areas at specific wavelengths: 400 nm (chlorophyll a), 470 nm (chlorophyll b), 286 nm (phytoene), 348 nm (phytofluene), and 450 nm (other carotenoids), with concentrations calculated against authentic standards.

### 2.3. Transmission Electron Microscopy (TEM)

Sample preparation and transmission electron microscopy were conducted as described in our previous study [20]. Plant samples were separated and fixed overnight at 4 °C in 2.5% glutaraldehyde (in 0.1 M phosphate buffer, pH 7.0). After fixation, samples were washed three times (15 min each) with phosphate buffer, then post-fixed in 1% OsO_4_ (in 0.1 M phosphate buffer, pH 7.0) for 1–2 h. Subsequently, samples were washed three times with phosphate buffer. Dehydration was performed using a graded ethanol series (50%, 70%, 80%, 90%, 95%, and 100%; 15 min per step), followed by infiltration in absolute acetone for 20 min. For resin infiltration, samples were sequentially treated with a 1:1 mixture of absolute acetone and Spurr resin for 1 h, a 1:3 mixture of absolute acetone and Spurr resin for 3 h, and finally, pure Spurr resin overnight. The embedded samples were placed in capsules filled with embedding medium and polymerized at 70 °C for 9 h. Prior to observation, ultrathin sections were stained with uranyl acetate and alkaline lead citrate (15 min each) and examined using a Hitachi JEM-1230 transmission electron microscope (Hitachi, Ltd., Tokyo, Japan).

### 2.4. RNA Extraction, RNA-Seq Analysis, and Quantitative Real-Time PCR (qPCR) Analysis

Total RNA was extracted by the CTAB method as described in our previous work [21]. High-quality total RNA was extracted, with RNA Integrity Numbers (RIN) consistently exceeding 7.0, as determined by Bioanalyzer analysis (Agilent Technologies, Santa Clara, CA, USA). RNA sequencing was performed by Biomarker Technologies on the Illumina HiSeq PE150 platform (Illumina, San Diego, CA, USA). For quantitative real-time PCR (qPCR) validation, first-strand cDNA was synthesized from 1 µg total RNA using the HiScript II 1st Strand cDNA Synthesis Kit (Vazyme, Nanjing, China). qPCR reactions were performed on a CFX96 Real-Time PCR Detection System (Bio-Rad, Hercules, CA, USA) using SYBR Green qPCR Master Mix (MedChemExpress, Monmouth Junction, NJ, USA), strictly adhering to manufacturer protocols. The thermal cycling profile consisted of (1) initial denaturation: 95 °C for 5 min; (2) 45 amplification cycles: 95 °C for 5 s, 58 °C for 15 s, and 72 °C for 10 s; and (3) melting curve analysis: 65 °C to 95 °C (increment 0.5 °C/5 s). Melting curve analysis confirmed amplification specificity for all reactions. The four carotenoid biosynthetic structural genes—*CsPSY1*, *CsPDS*, *CsZISO*, and *CsBCH*—were selected for qPCR validation of RNA-seq data accuracy. Transcript levels were normalized against the *CsActin* reference gene by the value of 2^−ΔCT^. Primers for RNA-seq validation were designed based on sequences of selected genes (Table S1).

### 2.5. Weighted Gene Co-Expression Network Analysis (WGCNA) and Establishment of Co-Expression Networks

Weighted Gene Co-expression Network Analysis (WGCNA) was implemented using the WGCNA R package v1.72 [23]. An adjacency matrix was constructed by raising the pairwise Pearson correlation matrix of all expressed genes to a soft-thresholding power (β = *X*, selected based on the scale-free topology criterion). This adjacency matrix was subsequently transformed into a Topological Overlap Matrix (TOM) to minimize spurious connections and measure network interconnectedness. Module identification was performed using average linkage hierarchical clustering of genes based on TOM dissimilarity (1-TOM). Modules were delineated from the resulting dendrogram using the dynamic tree-cutting algorithm. Branches of the dendrogram were assigned color codes representing distinct co-expression modules. Module-trait association analysis correlated module eigengenes (the first principal component of module expression) with phenotypic traits. Associations were visualized in a heatmap where rows represent modules (color-coded) and columns represent traits. Heatmap color intensity corresponds to the Pearson correlation coefficient (*r*) between module eigengene and trait (scale bar: red = positive correlation, blue = negative correlation, and white = |*r*| ≈ 0). Intramodular analysis identified highly connected genes (hub genes) within modules using connectivity measures. Network visualization for selected modules was performed using Cytoscape v3.10.3 [28]. Transcription factor (TF) identification was conducted by cross-referencing module gene lists with the Plant Transcription Factor Database (PlantTFDB; http://planttfdb.gao-lab.org/, accessed on 1 June 2025) [29].

## 3. Results

### 3.1. Identification and Classification of CsSnRK, CsPYL, and CsPP2C Gene Families in Citrus sinensis

**Figure 2:**
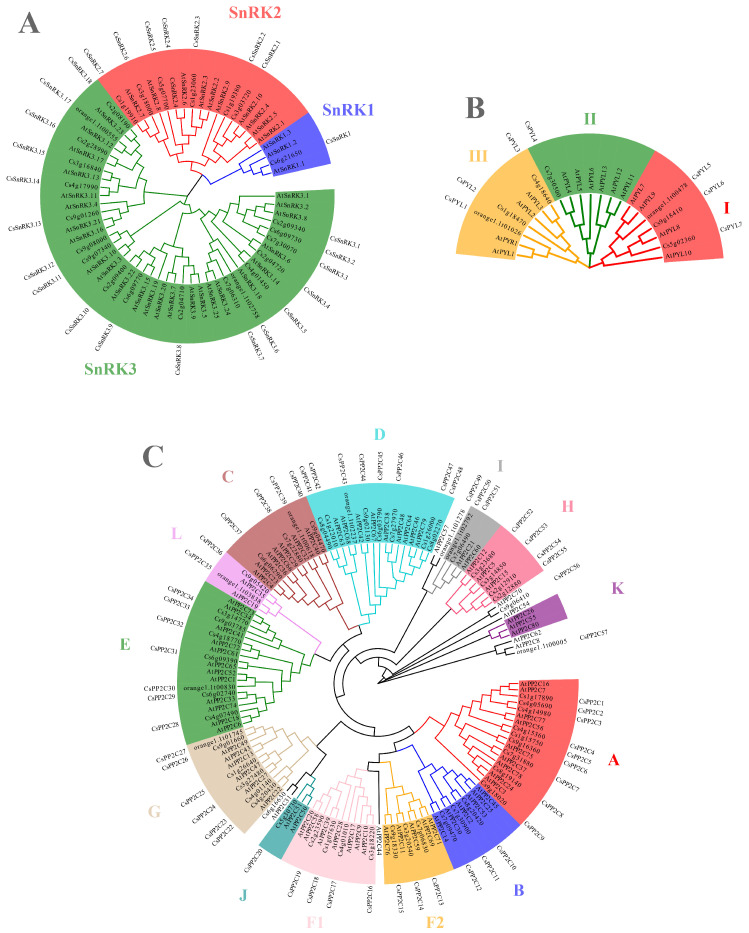
Phylogenetic analysis of SnRK, PYL, and PP2C families in *Citrus sinensis*. (**A**) SnRK family tree showing the three subfamilies SnRK1, SnRK2, and SnRK3. (**B**) PYL family tree showing the three subfamilies (I, II, III). (**C**) PYL family tree showing the 13 subfamilies (A–L). Trees were constructed using the neighbor-joining method in MEGA 7 with 1000 bootstrap replicates. Arabidopsis SnRKs, PYLs, and PP2Cs were included as references for identification and classification.

By integrating the HMMER-blastp-CDD pipeline, *CsSnRK*, *CsPYL*, and *CsPP2C* were identified (Figure 2). We identified 26 *CsSnRK* genes in *Citrus sinensis*, fewer than the 38 *AtSnRK* genes in Arabidopsis (Figure 2A). Based on subfamily classification [12], these comprise 1 *CsSnRK1*, 7 *CsSnRK2*, and 18 *CsSnRK3* genes, respectively fewer than the corresponding Arabidopsis subfamilies (3 *AtSnRK1*, 10 *AtSnRK2*, and 25 *AtSnRK3*) (Figure 2A). We also identified 7 *CsPYL* genes in sweet orange, indicating a contraction of this gene family compared to the 14 *AtPYL* genes in Arabidopsis (Figure 2B). Following established subfamily classification methods [17], the *CsPYL*s were grouped into three subfamilies (I, II, III): *CsPYL5*, *CsPYL6*, and *CsPYL7* belong to subfamily I; *CsPYL4* belongs to subfamily II; and *CsPYL1*, *CsPYL2*, and *CsPYL3* belong to subfamily III (Figure 2B). Furthermore, we identified 57 *CsPP2C* genes in sweet orange, fewer than the 80 *AtPP2C* genes in Arabidopsis (Figure 2C). Consistent with the established classification for the model plant Arabidopsis [19], these were categorized into 13 subfamilies (A, B, C, D, E, F1, F2, G, H, I, J, K, and L) (Figure 2C). A small number of genes remained unclassified into these subfamilies.

### 3.2. Comparative Profiling Reveals ‘Cara Cara’ Juice Vesicles Hyperaccumulate Linear Carotenoids (Phytoene, Phytofluene, and Lycopene) over ‘Newhall’, with Parallel Leaf/Flower Phytoene Elevation and Supporting Plastid Ultrastructure

**Figure 3:**
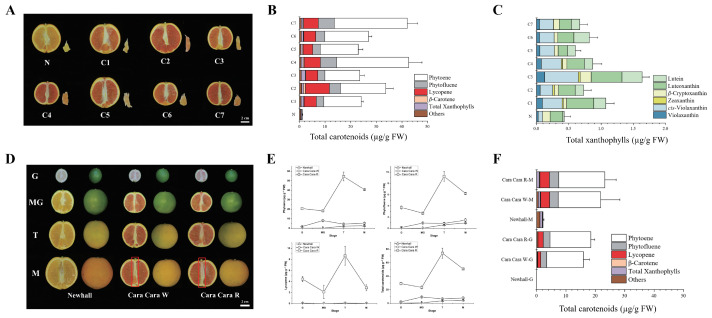
Morphological and carotenoid composition differences between ‘Newhall’ and ‘Cara Cara’ oranges exhibiting distinct color variation. (**A**) Longitudinal sections of mature ‘Newhall’ (N) and ‘Cara Cara’ (C1–C7) fruits. (**B**,**C**) Total carotenoid (**B**) and total xanthophyll (**C**) content in juice vesicles of individual fruits (fresh weight, FW). (**D**) Morphology of ‘Newhall’ and ‘Cara Cara’ fruits with white (W)- or red (R)-pigmented central cores at key developmental stages: green (G), mature Green (MG), turning (T), and mature (M). (**E**) Composition of carotenoids accumulated in red central cores (R), predominantly linear carotenoids (phytoene, phytofluene, and lycopene) with phytoene as the major component. (**F**) Total carotenoid content in juice vesicles (FW) of ‘Cara Cara’ fruits with white (W)- or red (R)-pigmented central cores across developmental stages. All data were derived from three independent biological replicates (*n* = 3), and error bars represent the standard error (SE).

Visual observation revealed uneven pigmentation and significant inter-fruit variability in the juice vesicles of ‘Cara Cara’ oranges. To document this phenomenon during fruit maturation, eight ‘Cara Cara’ fruits and one ‘Newhall’ fruit (as a control) were randomly sampled, as shown in Figure 3A. Carotenoid profiling of ‘Cara Cara’ juice vesicles confirmed high inter-fruit variability and revealed disconnected relationships between the color of the central core and its pigment composition (Figure 3B). The average total carotenoid content in ‘Cara Cara’ juice vesicles ranged from 22.91 to 42.61 µg/g FW, significantly higher than the 1.12 µg/g FW found in ‘Newhall’ (Figure 3B). Linear carotenoids (phytoene, phytofluene, and lycopene) dominated the total carotenoids in ‘Cara Cara’, accounting for 89.58% to 96.12%. Phytoene was the predominant component, constituting 52.42% to 67.60% of the total. In contrast, linear carotenoids represented only 16.07% of the total carotenoids in ‘Newhall’ juice vesicles, with phytoene at 11.31% (Figure 3B). While the average total xanthophylls in ‘Cara Cara’ juice vesicles were higher than in ‘Newhall’, no clear obligatory link existed between total xanthophyll levels and total carotenoid content (Figure 3C). For instance, Sample C3 had lower total carotenoids but higher xanthophylls, Sample C4 had the highest total carotenoids but lower xanthophylls, and Samples C1 and C3 had similar total carotenoid levels, but C3 had significantly more xanthophylls than C1 (Figure 3B,C).

**Figure 4:**
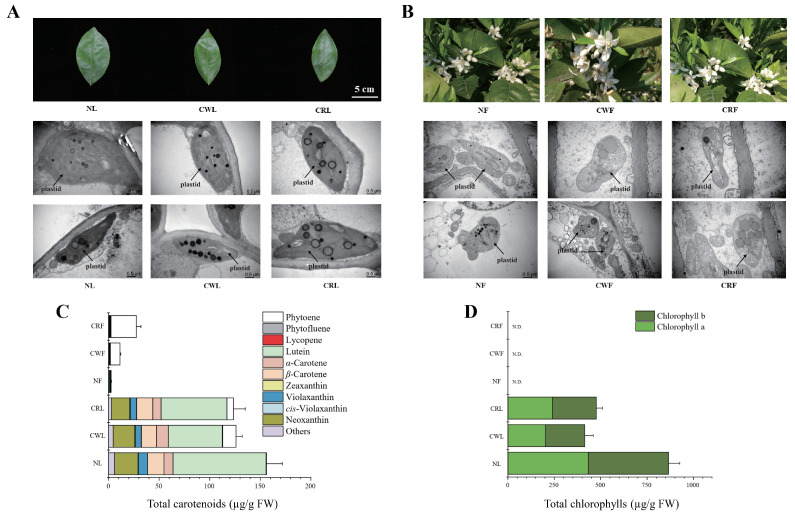
Comparative analysis of morphology, plastid ultrastructure, and pigment content in leaves and flowers of ‘Newhall’ and ‘Cara Cara’ oranges with white- or red-pigmented central cores. (**A**,**B**) Morphology and chloroplast ultrastructure in leaves (**A**: ‘Newhall’ leaf, NL; ‘Cara Cara’ white-core leaf, CWL; and ‘Cara Cara’ red-core leaf, CRL) and flowers (**B**: ‘Newhall’ flower, NF; ‘Cara Cara’ white-core flower, CWF; and ‘Cara Cara’ red-core flower, CRF). (**C**,**D**) Total carotenoid (**C**) and total chlorophyll (**D**) content in leaves and flowers of ‘Newhall’ and ‘Cara Cara’ oranges with white- or red-pigmented cores. All data were derived from three independent biological replicates (*n* = 3), and error bars represent the standard error (SE).

Longitudinal sectioning of individual ‘Cara Cara’ fruits revealed two distinct central core phenotypes: red (C3, C4, and C5) and white (C1, C2, C6, and C7) (Figure 3A). Subsequent developmental stage sampling confirmed that central core coloration is tree-specific; individual trees exclusively produce either red- or white-core fruits, indicating two distinct subpopulations within the ‘Cara Cara’ group (Figure 3D). This phenotypic divergence was observable as early as the immature green stage (Figure 3D). Red central cores accumulated lycopene (red pigment) alongside substantial colorless carotenoids (phytoene and phytofluene), mirroring the pigment profile of ‘Cara Cara’’s red juice vesicles (Figure 3E). However, central core coloration did not directly correlate with significant differences in juice vesicle carotenoid composition (Figure 3F). Comparative analysis of plant morphology showed minimal distinctions in leaf/flower (Figure 4A,B). Plastid ultrastructure among red-core ‘Cara Cara’, white-core ‘Cara Cara’, and ‘Newhall’ controls aligned with their carotenoid accumulation patterns (Figure 4B,C). Notably, both red- and white-core ‘Cara Cara’ leaves accumulated phytoene + phytofluene (6.44–13.54 µg/g FW, predominantly phytoene (>97%)), constituting 5.2–10.75% of total leaf carotenoids with no lycopene detected, while ‘Newhall’ leaves contained only trace phytoene (0.38 µg/g FW; 0.24% of total carotenoids) (Figure 4C); ‘Cara Cara’ flowers (both types) exhibited enlarged plastoglobuli consistent with phytoene hyperaccumulation and contained high phytoene + phytofluene (9.57–25.34 µg/g FW, >98% phytoene; 84.46–92.16% of total carotenoids), whereas ‘Newhall’ flowers showed minimal phytoene (0.28 µg/g FW; 8.21%) (Figure 4A–C). In contrast, ‘Newhall’ leaves had significantly higher chlorophyll content than ‘Cara Cara’ leaves, though chlorophyll levels did not differ between red- and white-core ‘Cara Cara’ (Figure 4D). In conclusion, beyond carotenoid profiles, ‘Newhall’ and ‘Cara Cara’ share highly similar physiological and morphological traits, establishing them as a suitable comparative system for investigating post-transcriptional regulation (e.g., SnRK, PYL, PP2C) of carotenoid biosynthesis.

### 3.3. Spatiotemporal Expression Dynamics of PP2C, SnRK, and PYL Gene Families Across Tissues and Fruit Developmental Stages Between ‘Newhall’ and ‘Cara Cara’

**Figure 5:**
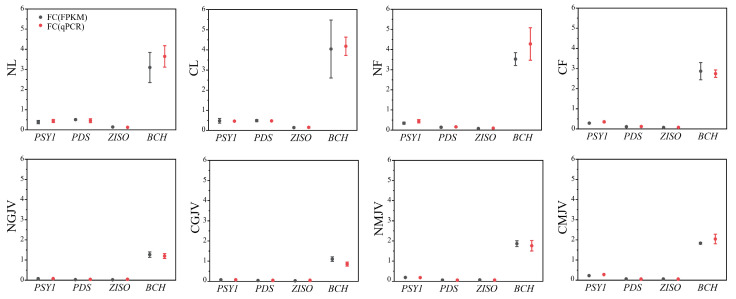
Validation of RNA-seq expression data by qPCR for key carotenoid biosynthetic genes. Expression levels of *CsPSY1*, *CsPDS*, *CsZISO*, and *CsBCH* were measured by both RNA-seq (FPKM) and qPCR (2^−ΔCT^) across various tissues and developmental stages. The relative fold-change values were normalized to *CsActin*. The strong concordance between the two methods confirms the reliability of the transcriptomic data. Tissue codes: NL, ‘Newhall’ leaf; CL, ‘Cara Cara’ leaf; NF, ‘Newhall’ flower; CF, ‘Cara Cara’ flower; NGJV, ‘Newhall’ green stage juice vesicle; CGJV, ‘Cara Cara’ green stage juice vesicle; NMJV, ‘Newhall’ mature stage juice vesicle; and CMJV, ‘Cara Cara’ mature stage juice vesicle. All data were derived from three independent biological replicates (*n* = 3), and error bars represent the standard error (SE).

**Figure 6:**
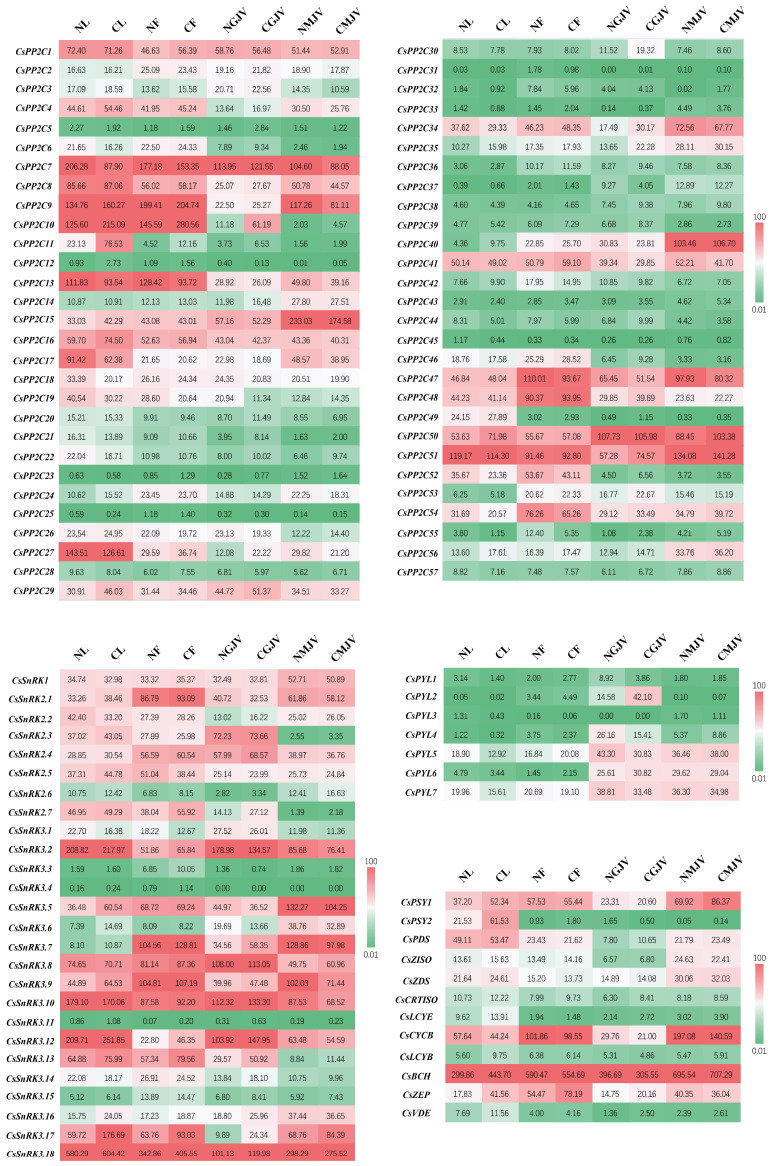
Heatmap of FPKM expression levels for *CsPP2C*s, *CsSnRK*s, *CsPYL*s, and carotenoid biosynthetic genes across tissues of ‘Newhall’ and ‘Cara Cara’ oranges. NL, ‘Newhall’ leaf; CL, ‘Cara Cara’ leaf; NF, ‘Newhall’ flower; CF, ‘Cara Cara’ flower; NGJV, ‘Newhall’ green stage juice vesicle; CGJV, ‘Cara Cara’ green stage juice vesicle; NMJV, ‘Newhall’ mature stage juice vesicle; and CMJV, ‘Cara Cara’ mature stage juice vesicle. To clearly visualize the expression differences of most genes, the color scale of the heatmap was set to a range of 0.01 to 100 (FPKM). Expression values beyond these limits are represented by the maximum or minimum color.

**Figure 7:**
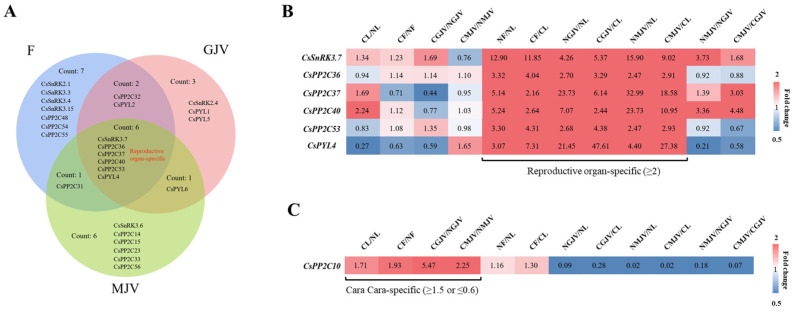
Reproductive organ-specific and ‘Cara Cara’-specific expression features of *CsSnRK*, *CsPP2C*, and *CsPYL* genes. (**A**) Venn diagram identifying reproductive organ-specific *CsSnRK*/*PP2C*/*PYL* genes. (**B**) Heatmap of developmental-stage expression profiles for reproductive organ-specific genes. (**C**) *CsPP2C10* as the sole ‘Cara Cara’-specific gene member and its developmental-stage expression heatmap. Expression specificity codes: F (floral-specific), GJV (green juice vesicle-specific), MJV (mature juice vesicle-specific). Tissue codes: NL (‘Newhall’ leaf), CL (‘Cara Cara’ leaf), NF (‘Newhall’ flower), CF (‘Cara Cara’ flower), NGJV (‘Newhall’ green juice vesicle), CGJV (‘Cara Cara’ green juice vesicle), NMJV (‘Newhall’ mature juice vesicle), and CMJV (‘Cara Cara’ mature juice vesicle). To clearly visualize the expression differences of most genes, the color scale of the heatmap was set to a range of 0.5 to 2 (fold change). Expression values beyond these limits are represented by the maximum or minimum color.

qPCR validation of four carotenoid-biosynthetic structural genes (*CsPSY1*, *CsPDS*, *CsZISO*, and *CsBCH*) confirmed consistency with RNA-seq data, verifying the reliability of transcriptomic analyses (Figure 5). Given minimal carotenoid differences between red- and white-core ‘Cara Cara’, equal-weight pooled samples from both phenotypes were used to represent ‘Cara Cara’ versus ‘Newhall’. FPKM-based expression heatmaps of *PP2C*, *SnRK*, and *PYL* families across tissues and developmental stages revealed (1) constitutively high expression of *CsPP2C1*/*7*/*50*/*51* and *CsSnRK1*/*2.4*/*3.2*/*3.8*/*3.9*/*3.10*/*3.18*, and (2) consistently low/absent expression of *CsPP2C12*/*23*/*25*/*31*/*45*, *CsSnRK3.4*/*3.11*, and *CsPYL3* (the sole low-expressing *PYL* member) (Figure 6). Key carotenoid structural genes showed comparable expression in ‘Newhall’ and ‘Cara Cara’ (Figure 6), failing to explain ‘Cara Cara’’s > 26-fold higher phytoene accumulation in leaves, flowers, and juice vesicles (Figure 3B,F and Figure 4C). Tissue-specific genes were filtered using the following thresholds: floral-specific (F: NF/NL ≥ 2 & CF/CL ≥ 2), young fruit juice vesicle-specific (GJV: NGJV/NL ≥ 2 & CGJV/CL ≥ 2), and mature fruit juice vesicle-specific (MJV: NMJV/NL ≥ 2 and CMJV/CL ≥ 2) (Table 1). Venn analysis identified six reproductive organ-specific genes (*CsSnRK3.7*, *CsPP2C36*/*37*/*40*/*53*, and *CsPYL4*) potentially regulating fruit maturation and carotenoid metabolism (Figure 7). Screening for ‘Cara Cara’-specific regulators (‘Cara Cara’/’Newhall’ ≥ 1.5 or ≤0.6) pinpointed a single candidate, *CsPP2C10*, providing a critical lead for investigating phytoene hyperaccumulation (Figure 7).

### 3.4. WGCNA Integrates Transcriptome and Carotenoid Profiles Revealing a ‘Cara Cara’-Specific Module for Linear Carotenoid Accumulation; Network Analysis Identifies Candidate SnRK, PYL, PP2C, and TF Regulators of Post-Transcriptional Control

**Figure 8:**
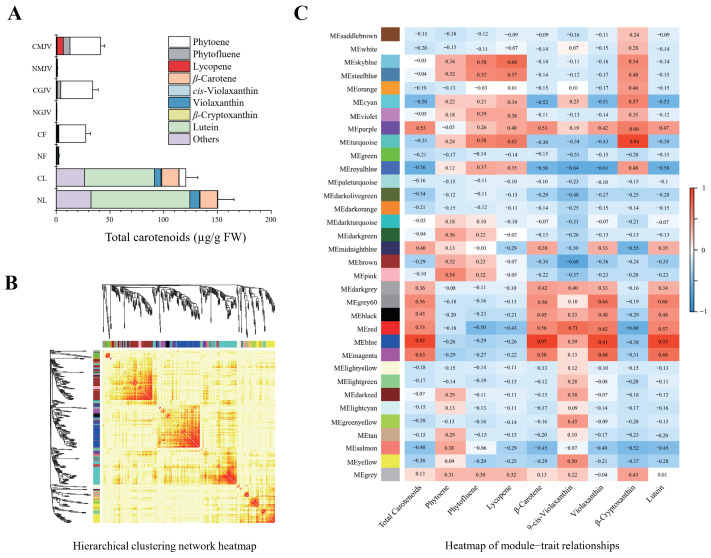
Integrated WGCNA of transcriptome and carotenoid metabolism across tissues and developmental stages. (**A**) Carotenoid metabolite profiles in sampled tissues. (**B**) Hierarchical clustering of co-expression modules. (**C**) Module-trait relationship heatmap; heatmap values indicate correlation coefficients between modules and specific carotenoids. Tissues: NL (‘Newhall’ leaf), CL (‘Cara Cara’ leaf), NF (‘Newhall’ flower), CF (‘Cara Cara’ flower), NGJV (‘Newhall’ green juice vesicle), CGJV (‘Cara Cara’ green juice vesicle), NMJV (‘Newhall’ mature juice vesicle), and CMJV (‘Cara Cara’ mature juice vesicle).

To investigate the potential regulation of *CsSnRK*, *CsPP2C*, and *CsPYL* gene families on carotenoid biosynthesis, specifically phytoene hyperaccumulation in ‘Cara Cara’, we performed WGCNA co-expression analysis integrating transcriptome data from ‘Cara Cara’ and ‘Newhall’ tissues from different organs and fruit developmental stages with carotenoid metabolic profiles (Figure 8A), aiming to identify modules associated with specific carotenoid accumulation (Figure 8).

Hierarchical clustering and module-trait heatmaps defined 34 distinct modules, each characterized by unique carotenoid accumulation patterns (Figure 8B,C). Focusing on modules linked to phytoene, phytofluene, and lycopene accumulation in ‘Cara Cara’ juice vesicles, we ranked modules by summing correlation coefficients for these three traits; the sky-blue, turquoise, and steel-blue modules topped the list (Table 2).

**Figure 9:**
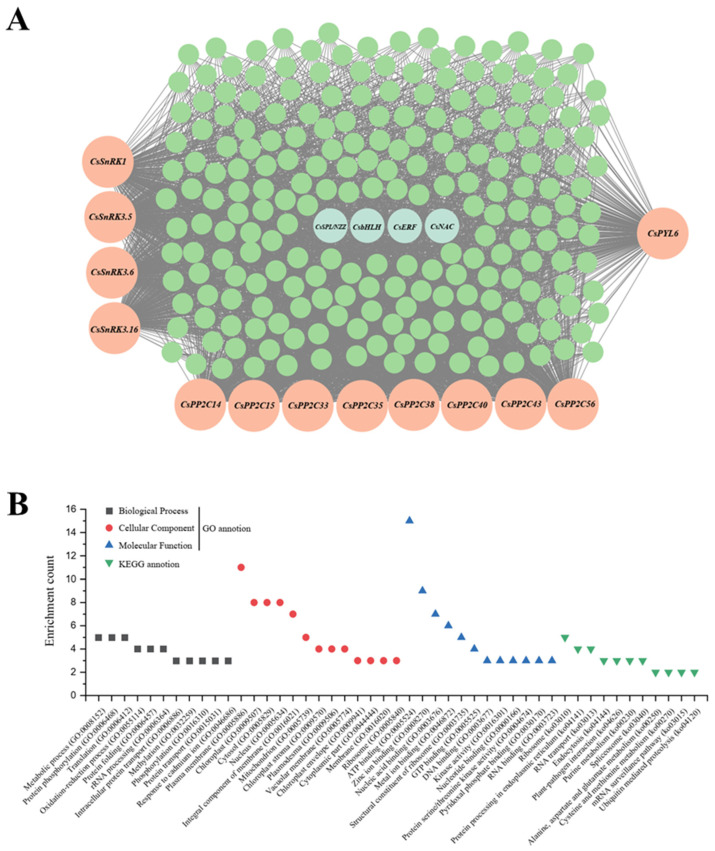
Co-expression network and functional enrichment analysis of the turquoise module. (**A**) Gene–gene interaction network visualized with Cytoscape. (**B**) Functional enrichment of GO terms and KEGG pathways.

Analysis of *CsSnRK*/*PP2C*/*PYL* family distribution across modules revealed turquoise contained the highest number (13 members: *CsSnRK1*/*3.5*/*3.6*/*3.16*, *CsPP2C14*/*15*/*33*/*35*/*38*/*40*/*43*/*56*, and *CsPYL6*) (Table 3) alongside key carotenoid structural genes (*CsPSY1*, *CsZISO*, and *CsZDS*), implicating turquoise in regulating phytoene/phytofluene/lycopene synthesis (Table 4, Tables S2 and S3; Figure S4). Within this module, four transcription factors (*SPL*/*NZZ*, *NAC*, *bHLH*, and *ERF*) potentially bridge post-transcriptional phosphorylation to transcriptional control of structural genes. Connectivity analysis prioritized regulatory hubs: *SPL*/*NZZ* and *bHLH* ranked highly, while structural genes (*CsPSY1*/*ZISO*/*ZDS*; ranks 2332/2082/1847) showed low connectivity as expected; notably, *CsSnRK1* and *CsPP2C14*/*15*/*40*/*56* exhibited high connectivity, with *CsPP2C40* (rank 67) emerging as a potential hub gene (Table 4). Cytoscape-visualized co-expression networks revealed gene interactions (Figure 9A, Table S4). GO/KEGG enrichment of turquoise highlighted top terms: metabolic process (GO:0008152), protein phosphorylation (GO:0006468), plasma membrane (GO:0005886), chloroplast (GO:0009507), ATP binding (GO:0005524), and pathways including Ribosome (ko03010), Protein processing in endoplasmic reticulum (ko04141), and RNA transport (ko03013)—indicating processes relevant to carotenoid metabolism and post-transcriptional regulation (Figure 9B).

## 4. Discussion

This study provides a comprehensive genomic identification of the *SnRK*, *PYL*, and *PP2C* families in sweet orange and leverages the natural phenotypic variation between the wild-type ‘Newhall’ and the ‘Cara Cara’ mutant to investigate post-transcriptional regulatory mechanisms controlling carotenoid metabolism. Multi-omics analysis serves as a powerful and indispensable tool in plant research, enabling the systematic discovery of novel regulatory mechanisms, key genes, and signaling metabolites that govern complex plant responses to development and environmental stresses [30,31,32,33,34]. Our multi-omics approach, integrating genomics, transcriptomics, metabolomics, and co-expression network analysis, yields several novel insights with significant implications for both basic plant biology and applied horticulture.

### 4.1. A Putative Phosphorylation Network Orchestrates Carotenoid Hyperaccumulation

The most salient finding of this study is the pronounced disconnect between transcript levels of carotenoid biosynthetic genes and the massive (26-fold) accumulation of linear carotenoids in ‘Cara Cara’ across tissues (leaves, flowers, and juice vesicles) (Figure 3, Figure 4 and Figure 6). This strongly implicates regulatory mechanisms acting post-transcriptionally. While post-transcriptional regulation is crucial for plant signal transduction, its role in metabolic synthesis is less explored; SnRK kinases emerge as pivotal core factors mediating this level of regulation [35,36,37,38,39]. Our WGCNA analysis identified a key turquoise module highly correlated with phytoene, phytofluene, and lycopene accumulation. The co-localization within this module of genes from the post-transcriptional cascade (*CsSnRK1*/*3.5*/*3.6*/*3.16*, *CsPP2C14*/*15*/*33*/*35*/*38*/*40*/*43*/*56*, and *CsPYL6*), key biosynthetic enzymes (*CsPSY1*, *CsZISO*, and *CsZDS*), and candidate transcription factors (*SPL*/*NZZ*, *NAC*, *bHLH*, and *ERF*) suggests an intricate regulatory network where phosphorylation is a central mechanism (Table 4, Figure 8).

This proposed network aligns with emerging evidence that post-translational modifications are crucial for metabolic regulation. For instance, *SnRK2*s are well-established kinases in ABA signaling that phosphorylate downstream targets like transcription factors and enzymes [12,13,15]. Our finding that specific *CsPP2C*s (e.g., *CsPP2C40*, a high-connectivity hub) and *CsSnRK*s are central to this module suggests a mechanism where SnRK-mediated phosphorylation and PP2C-mediated dephosphorylation fine-tune the activity of proteins involved in the carotenogenic pathway. This could occur through direct phosphorylation of biosynthetic enzymes, altering their stability or activity, as demonstrated for other metabolic pathways [40,41], or more likely indirectly through the phosphorylation of transcription factors that regulate structural gene expression [13,42,43,44,45].

### 4.2. Bridging ABA Signaling and Carotenoid Metabolism: A Potential Feedback Loop

A particularly intriguing aspect of our model is its connection to ABA signaling. ABA is a carotenoid-derived hormone, creating a potential feedback loop where the hormone regulates its own biosynthetic precursors. Our results suggest that components of the core ABA signaling pathway (PYL-PP2C-SnRK) are co-expressed with carotenogenic genes (Table 4, Figure 6 and Figure 8). This implies that the SnRK-PP2C-PYL network in citrus may not only respond to ABA but also directly regulate carotenoid precursor availability.

This finding adds a new layer to the understanding of ABA’s role beyond stress response, positioning it as a direct modulator of core metabolism. It would be highly relevant to explore how environmental stresses known to induce ABA production (e.g., drought) impact carotenoid accumulation in these genotypes via this proposed mechanism, thus bridging the gap between the abstract’s mention of stress resilience and the discussion of metabolic adaptation.

### 4.3. Scientific Advance and Comparison with Previous Studies

**Figure 10:**
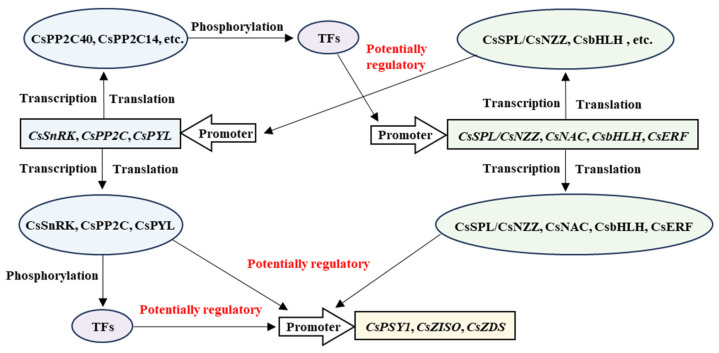
Putative dual-layer regulatory network mediated by SnRK/PP2C/PYL in carotenoid metabolism. Integrates transcript-level (TF-mediated) and post-transcriptional (phosphorylation) control. Diagram elements: arrows = promoters; rectangles = genes; ovals = proteins.

Our work represents a significant advance over previous studies in citrus [22]. While earlier research identified some *SnRK2* and *PP2C* members, our study provides the first comprehensive genome-wide analysis of all three gene families (*SnRK*, *PYL*, and *PP2C*) in sweet orange, revealing a contraction of the *CsSnRK*, *CsPP2C*, and *CsPYL* families compared to Arabidopsis (Figure 2). More importantly, by moving beyond descriptive gene identification and employing WGCNA on multi-tissue, multi-omics data from contrasting genotypes, we predict a functional, post-transcriptional regulatory network (Figure 10). This approach addresses a critical gap in the field, as the regulatory mechanisms controlling carotenoid accumulation in citrus, particularly post-transcriptionally, have remained largely unexplored.

### 4.4. Limitations and Future Perspectives

It is crucial to highlight that the regulatory model proposed in Figure 10, while informed by robust co-expression analysis, remains speculative and requires direct experimental validation. The arrows labeled “potentially regulatory” denote predicted interactions based on correlation and connectivity, not confirmed mechanistic links. A major limitation of this study is the lack of functional validation experiments. Future studies must include phosphorylation assays (e.g., in vitro kinase assays, phosphoproteomics), protein–protein interaction studies (e.g., Y2H, Co-IP, BiFC) to confirm the interactions between CsSnRKs/CsPP2Cs and their putative targets (e.g., *CsPSY1*, TFs), and functional characterization through gene overexpression/knockdown in citrus or model systems to assess the impact on carotenoid profiles.

Furthermore, the exploration of the relationship between plant resilience and hormonal regulation, as mentioned in the abstract, is not fully developed here. Future work should explicitly test whether the proposed network confers enhanced stress resilience to the ‘Cara Cara’ mutant by subjecting both genotypes to abiotic stresses and monitoring changes in the network components and carotenoid accumulation.

## 5. Conclusions

In conclusion, our study moves beyond transcriptional regulation and provides compelling evidence for a novel, phosphorylation-mediated post-transcriptional mechanism that fine-tunes carotenoid accumulation in citrus. We propose a model where an expanded SnRK-PP2C-PYL network, integrated with transcriptional regulators, modulates carotenogenic flux, potentially linking ABA signaling to metabolic homeostasis. This work not only provides a foundational resource of candidate genes but also unveils a new regulatory layer for carotenoid metabolism, offering prioritized molecular targets for breeding nutrient-dense and climate-resilient citrus varieties. The validation of this proposed network constitutes an essential and exciting direction for future research.

## Figures and Tables

**Table 1:** Stage-specific expression patterns of *CsSnRK*, *CsPP2C*, and *CsPYL* genes during fruit development in ‘Newhall’ and ‘Cara Cara’ oranges. Expression specificity codes: F (floral-specific), GJV (green juice vesicle-specific), and MJV (mature juice vesicle-specific). Tissue codes: NL (‘Newhall’ leaf), CL (‘Cara Cara’ leaf), NF (‘Newhall’ flower), CF (‘Cara Cara’ flower), NGJV (‘Newhall’ green juice vesicle), CGJV (‘Cara Cara’ green juice vesicle), NMJV (‘Newhall’ mature juice vesicle), and CMJV (‘Cara Cara’ mature juice vesicle).

F Expressed Genes		GJV Expressed Genes		MJV Expressed Genes	
*CsSnRK2.1*; *CsSnRK3.3*; *CsSnRK3.4*; *CsSnRK3.7*; *CsSnRK3.15*	NF/NL≥2 and CF/CL≥2	*CsSnRK2.4*; *CsSnRK3.7*	NGJV/NL≥2 and CGJV/CL≥2	*CsSnRK3.6*; *CsSnRK3.7*	NMJV/NL≥2 and CMJV/CL≥2
*CsPP2C31*; *CsPP2C32*; *CsPP2C36*; *CsPP2C37*; *CsPP2C40*; *CsPP2C48*; *CsPP2C53*; *CsPP2C54*; *CsPP2C55*		*CsPP2C36*; *CsPP2C37*; *CsPP2C40*; *CsPP2C53*		*CsPP2C14*; *CsPP2C15*; *CsPP2C23*; *CsPP2C31*; *CsPP2C33*; *CsPP2C36*; *CsPP2C37*; *CsPP2C40*; *CsPP2C53*; *CsPP2C56*	
*CsPYL2*; *CsPYL4*		*CsPYL1*; *CsPYL2*; *CsPYL5*; *CsPYL6*		*CsPYL4*; *CsPYL6*	

**Table 2:** Correlation coefficients of WGCNA modules with specific carotenoids (phytoene, phytofluene, and lycopene) and cumulative ranking.

Module	Phytoene	Phytofluene	Lycopene	Sum	Rank
MEskyblue	0.34	0.58	0.68	1.60	1
MEturquoise	0.24	0.58	0.63	1.45	2
MEsteelblue	0.32	0.52	0.57	1.41	3
MEviolet	0.18	0.39	0.38	0.95	4
MEgrey	0.31	0.30	0.32	0.93	5
MEroyalblue	0.12	0.37	0.35	0.84	6
MEcyan	0.22	0.21	0.34	0.77	7

**Table 3:** Distribution frequency of *CsSnRK*, *CsPP2C*, and *CsPYL* genes across WGCNA co-expression modules. Values indicate gene counts and percentage representation per module (sorted by frequency).

Module	Count	Percentage
turquoise	13	13.48%
black	10	11.24%
red	10	11.24%
blue	9	10.11%
yellow	9	10.11%
brown	6	6.74%
cyan	6	6.74%
purple	5	5.62%
royalblue	5	5.62%
salmon	4	4.49%
grey60	3	3.37%
midnightblue	2	2.25%
darkolivegreen	1	1.12%
darkred	1	1.12%
greenyellow	1	1.12%
lightcyan	1	1.12%
lightgreen	1	1.12%
lightyellow	1	1.12%
magenta	1	1.12%
pink	1	1.12%

**Table 4:** Intramodular connectivity and ranking of hub genes in the turquoise co-expression module. Genes include members of the *CsSnRK*, *CsPP2C*, and *CsPYL* families, transcription factors, and carotenoid-biosynthetic structural genes. Connectivity represents node connectivity in the scale-free network, where higher values indicate greater potential for regulatory interactions and elevated hierarchical importance (sorted by rank).

Classification	Gene Name	Gene ID	Connectivity	Rank by Connectivity
**SnRK**	*CsSnRK1*	Cs6g21650	750.35	602
	*CsSnRK3.5*	Cs4g01450	102.98	3788
	*CsSnRK3.6*	orange1.1t02758	522.88	1331
	*CsSnRK3.16*	Cs2g28990	582.86	1093
				
**PP2C**	*CsPP2C14*	Cs3g20540	971.09	106
	*CsPP2C15*	Cs9g18330	796.31	496
	*CsPP2C33*	Cs9g03785	455.52	1578
	*CsPP2C35*	orange1.1t03838	337.26	2119
	*CsPP2C38*	Cs7g22880	303.26	2288
	*CsPP2C40*	Cs9g04470	998.44	67
	*CsPP2C43*	orange1.1t02237	230.55	2780
	*CsPP2C56*	Cs9g06410	824.48	433
				
**PYL**	*CsPYL6*	Cs9g18410	348.99	2060
				
**Transcription factor**	*SPOROCYTELESS*/*NOZZLE* (*SPL*/*NZZ*)	Cs1g06080	705.83	711
	*NAC*	Cs1g09660	99.36	3825
	*bHLH*	Cs1g02580	612.51	995
	*AP2-EREBP* (*ERF*)	Cs1g03300	164.06	3236
				
**Carotenoid biosynthesis structural genes**	*CsPSY1*	Cs6g15910	295.83	2332
	*CsZISO*	Cs5g24730	343.93	2082
	*CsZDS*	Cs3g11180	396.55	1847

## Data Availability

The data presented in this study are available on request from the corresponding author. The data are not publicly available due to privacy.

## Supplementary Materials

The following supporting information can be downloaded at https://www.mdpi.com/article/10.3390/metabo15090610/s1: Figure S1: Protein sequences of CsSnRKs and AtSnRKs for phylogenetic analysis; Figure S2: Protein sequences of CsPP2Cs and AtPP2Cs for phylogenetic analysis; Figure S3: Protein sequences of CsPYLs and AtPYLs for phylogenetic analysis; Figure S4: Amino acid sequences derived from predicted ORFs of 222 genes in the turquoise module; Table S1: Primer for qPCR; Table S2: All genes in turquoise module; Table S3: turquoise 222 genes annotation; Table S4: Network analysis in Cytoscape.

## Abbreviations

The following abbreviations are used in this manuscript:

SnRK	sucrose non-fermenting related kinases
PYR1	pyrabactin resistance
PYL	PYR1-like
RCAR	regulatory components of ABA receptors
PP2C	protein phosphatases type 2C
TFs	transcription factors
GGPP	geranylgeranyl pyrophosphate
PSY	phytoene synthase
PDS	phytoene desaturase
Z-ISO	ζ-carotene isomerase
ZDS	ζ-carotene desaturase
CrtISO	carotene isomerase
CYCB	chromoplast-specific lycopene β-cyclase
LCYB	lycopene β-cyclase
LCYE	lycopene ε-cyclase
BCH	β-carotene hydroxylase
ZEP	zeaxanthin epoxidase
VDE	violaxanthin de-epoxidase
NSY	neoxanthin synthase
NCED	9-cis-epoxycarotenoid dioxygenase
CCD	carotenoid cleavage dioxygenase
NL	‘Newhall’ leaf
CL	‘Cara Cara’ leaf
NF	‘Newhall’ flower
CF	‘Cara Cara’ flower
NGJV	‘Newhall’ green stage juice vesicle
CGJV	‘Cara Cara’ green stage juice vesicle
NMJV	‘Newhall’ mature stage juice vesicle
CMJV	‘Cara Cara’ mature stage juice vesicle
WGCNA	weighted gene co-expression network analysis
Co-IP	co-immunoprecipitation
BiFC	bimolecular fluorescence complementation
EMSA	electrophoretic mobility shift assays
qPCR	quantitative real-time PCR
