# Supplementary material for: SnRK-PP2C-PYL Gene Families in Citrus sinensis: Genomic Characterization and Regulatory Roles in Carotenoid Metabolism
Source: Metabolites. 2025 Sep 12;15(9):610. doi: 10.3390/metabo15090610 (PMC12471423; doi:10.3390/metabo15090610)
Supplement: Supplementary file 1 [file metabolites-15-00610-s001.zip › Supplementary Table S1. Primer for qPCR.pdf]

Supplementary Table S1. List of primers for qPCR

Gene	Primers (Forward; Reverse)
CsActin (reference gene)	5'-CTACAGAGAGTTGAAGTTACAGGG-3'
	5'-TAGGGCATCGGCCTTGGATTGTG-3'
CsPSY1	5'-GGAATCGACTTGCCTGAAAT-3'
	5'-TGCCTGCTTGAGCACAACAT-3'
CsPDS	5'-TTCAGCCGATTTGATTTTCC-3'
	5'-ACACCCTGCTTTCTCATCCA-3'
CsZISO	5'-ACTCCGTCTCCTCCCTTTCA-3'
	5'-TTACCTGCCCAACCATCTGT-3'
CsBCH	5'-GTTTGCCATAATCAACGC-3'
	5'-CTCTCCGGAAATAAGGCA-3'
